# Optimizing Tactics for use of the U.S. Antiviral Strategic National Stockpile for Pandemic (H1N1) Influenza, 2009

**DOI:** 10.1371/currents.RRN1127

**Published:** 2009-11-04

**Authors:** Nedialko Dimitrov, Sebastian Goll, Lauren Ancel Meyers, Babak Pourbohloul, Nathaniel Hupert

**Affiliations:** ^*^The University of Texas at Austin; ^§^Division of Mathematical Modeling, British Columbia Centre for Disease Control and ^¶^Weill Cornell Medical College (NYC) and Preparedness Modeling Unit, U.S. Centers for Disease Control and Prevention (CDC, Atlanta)

## Abstract

Public health agencies across the globe are working to mitigate the impact of the 2009 pandemic caused by swine-origin influenza A (H1N1) virus. Prior to the large-scale distribution of an effective vaccine, the primary modes of control have included careful surveillance, social distancing and hygiene measures, strategic school closures, other community measures, and the prudent use of antiviral medications to prevent infection (prophylaxis) or reduce the severity and duration of symptoms (treatment). Here, we use mathematical models to determine the optimal geo-temporal tactics for distributing the U.S. strategic national stockpile of antivirals for treatment of infected cases during the early stages of a pandemic, prior to the wide availability of vaccines.

We present a versatile optimization method for efficiently searching large sets of public health intervention strategies, and apply it to evaluating tactics for distributing antiviral medications from the U.S. Strategic National Stockpile (SNS). We implemented the algorithm on a network model of H1N1 transmission within and among U.S. cities to project the epidemiological impacts of antiviral stockpile distribution schedules and priorities. The resulting optimized strategies critically depend on the rates of antiviral uptake and wastage (through misallocation or loss). And while a surprisingly simple pro rata distribution schedule is competitive with the optimized strategies across a wide range of uptake and wastage, other equally simple policies perform poorly.

Even as vaccination campaigns get underway worldwide, antiviral medications continue to play a critical in reducing H1N1-associated morbidity and mortality. If efforts are made to increase the fraction of cases treated promptly with antivirals above current levels, our model suggests that optimal use of the antiviral component of the Strategic National Stockpile may appreciably slow the transmission of H1N1 during fall 2009, thereby improving the impact of targeted vaccination. A more aggressive optimized antiviral strategy of this type may prove critical to mitigating future flu pandemics, but may increase the risk of antiviral resistance.

## Author Summary

The U.S. Government holds millions of treatment courses of antiviral medications its Strategic National Stockpile (SNS), but there are no published criteria for sequential release of these medications to States and Territories during the course of a worldwide influenza pandemic, such as the one currently caused by the pandemic influenza A (H1N1) virus.. We employed advanced optimization methods to evaluate millions of antiviral distribution schedules that vary in both the amount of each release and whether allocations to states are proportional to overall population or recent flu activity. We find that, for the case of 2009 (H1N1) pandemic flu, a simple distribution schedule of periodic small releases proportional to state population densities is a near-optimal policy. For this antiviral distribution scheme to have an impact on transmission of the current H1N1 virus, however, a higher proportion of cases must be identified and treated promptly with antivirals than is currently the case. This paper provides both practical guidance for future flu intervention and new methods that are readily adaptable to optimizing a broad range of infectious disease control policies.

## Introduction

In March/April 2009, a new swine-origin strain of influenza A/H1N1 virus emerged into human populations in California and Mexico and has since escalated into a pandemic. The U.S. experienced low levels of sustained H1N1 transmission throughout the summer months of 2009, and in the Southern hemisphere the novel virus largely displaced seasonal influenza, causing more severe morbidity in a younger adults and children than is typical with "the flu." Starting in September, the U.S. began to experience large geo-temporally distinct outbreaks of influenza like illness and confirmed H1N1 disease in States and Territories. As of mid-October, total cases reported by surveillance systems as well as pediatric deaths had surpassed levels typically seen for the entire flu season ending in mid-Spring 2010.

During the early weeks of the epidemic, the U.S. Centers for Disease Control and Prevention (CDC) distributed 11 million of the 50 million antiviral treatment courses from the federally held portion of the national antiviral stockpile. This distribution went to States and Territories (as well as to four large cities) on a pro rata basis (that is, in proportion to population size). Since the recipients had local stockpiles as well, this allowed the CDC to exceeded the pre-determined target of distribution of 31 million treatment courses of oseltamivir and zanamivir prior to the acceleration phase of the pandemic [1]. With additional purchases, the Federal stockpile now remains at approximately 50 million treatment courses, but there is no clear quantitative guidance to indicate the optimal use of these countermeasures.

Key policy statements have called for the use of mathematical models to support the development of an evidence-based policy for eﬀectively deploying the remaining antiviral stockpile and other limited or costly measures to limit morbidity and mortality from H1N1 [2] [3]. While mathematical modelers have taken great strides towards building predictive models of disease transmission dynamics within human populations, the computational complexity of these models often precludes systematic optimization of the demographic, spatial and temporal distribution of costly resources. Thus the typical approach has been to evaluate a relatively small set of candidate strategies [4] [5] [6] [7].

Here, we use a new algorithm that efficiently searches large strategy spaces to analyze the optimal use of the U.S. antiviral stockpile against pandemic influenza prior to widespread and effective vaccination. We assume, in line with CDC guidance, that antivirals will be used exclusively for treatment of symptomatic individuals rather than wide scale pre-exposure prophylaxis. We apply our algorithm to a U.S. national-scale network model of H1N1 transmission that is based on demographic and travel data from the U.S. Census Bureau and the Bureau of Transportation Statistics and assumes disease parameters estimated for H1N1 during the initial March-April 2009 outbreak in Mexico City.

## Methods

We couple a fast, scalable, and adaptable optimization algorithm to a detailed simulation model of influenza transmission within and among the 100 largest cities in the United States. In brief, the method involves running many stochastic simulations of H1N1 transmission, each requiring an intervention policy as an argument. The optimization algorithm dictates the choice of intervention policy for each simulation, with the goal of identifying the optimal intervention policy given the highly stochastic simulation output.

### Optimization method

A time-based intervention policy is a series of actions \begin{equation*}A_1, A_2, \ldots, A_D\end{equation*} taken in sequence over \begin{equation*}D\end{equation*} time periods (Fig. 1). Our objective is to rapidly search large sets of time-based intervention policies to ﬁnd those that will be most eﬀective at achieving a public health goal, such as limiting morbidity and mortality associated with inﬂuenza. To achieve this, we use trees to represent all possible policies (Fig. 1). The ﬁrst (highest) level of a policy tree is a single node attached to several edges; each of those edges corresponds to one of the possible actions in the first time period and leads to a level-two node. Similarly each level-two node is attached to edges corresponding to all possible actions during the second time period, and so on. Each intervention policy corresponds to a unique path through the tree.

**Figure:**
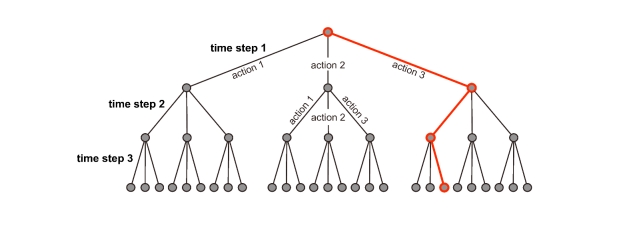
**Figure 1.** Simple policy tree: Suppose there are three possible actions and, in each time step, we can only choose one of them. Each level in the tree corresponds to a time step and branches represent possible actions. The red path through the tree represents the following three-step time-based intervention: First choose action 3, then action 1, and finally action 3 again. The policy tree for antiviral distribution has a similar organization. The UCT algorithm iteratively selects paths through the tree that represent intervention policies to be simulated.

The naive approach to finding the optimal path through the tree is to simulate multiple disease outbreaks for each intervention policy (path) and record the expected morbidity or mortality (or other public health outcome measure). However, such exhaustive searches are computationally intractable for large trees. We can more efficiently search for the optimal policy by prudently sampling paths from the tree.

To strategically search the tree, we use an optimization algorithm called Upper Confidence Bounds Applied to Trees (UCT) [8] [9]. It selects paths from the tree using a multi-armed bandit algorithm inside of each tree node. The canonical application of a bandit algorithm is maximizing the total payoff from playing a set of slot machines for a fixed number of rounds, where the payoff distributions of the machines are unknown and, in each round, we may select only one machine. In this scenario, each edge emanating from the node corresponds to a slot machine that can be chosen by the node’s bandit algorithm; for a policy tree, the edges correspond to possible policy actions. Before each policy simulation, bandit algorithms within the nodes select an edge to follow based on the results of prior trials. The combined choices of the bandit algorithms produce a path through the tree, corresponding to a sequence of public health actions, that is then passed into the simulation. The bandit algorithms determine which edge (action) to follow next by balancing two desirable characteristics: strong past performance and few prior trials. With this strategic pathsampling, subtrees with good performance are explored more thoroughly than those with poor performance.

Specifically, suppose we are descending through the tree and have arrived at node \begin{equation*}n\end{equation*} having \begin{equation*}k\end{equation*} edges to the next level down, \begin{equation*}(e_1, e_2, \ldots, e_k)\end{equation*}, representing all possible subsequent actions. Let \begin{equation*}N(e_i)\end{equation*} be the number of times we have used the intervention represented by edge \begin{equation*}e_i\end{equation*} in prior simulations and \begin{equation*}R(e_i)\end{equation*} be a real number between 0 and 1, describing the average rewards observed during past simulations where ei was chosen. In the analysis described below, the reward for a simulation is the fraction of individuals that remain uninfected during the outbreak. Now define \begin{equation*}V = \sum_{i=1}^{k} N(e_i)\end{equation*} to be the total number of times we have used descendants of \begin{equation*}n\end{equation*} in past simulations. We then select the next edge as given by

\begin{equation*}\operatorname{argmax}_e \left\{ R(e) + \sqrt{\frac{2 \cdot V}{N(e)}} \right\}\end{equation*}.

Initially, \begin{equation*}N(e)\end{equation*} and \begin{equation*}R(e)\end{equation*} are set to zero for each edge \begin{equation*}e\end{equation*}. The first \begin{equation*}k\end{equation*} times we arrive at node \begin{equation*}n\end{equation*}, we choose the next edge uniformly randomly from the previously unsampled edges descending from the node, rather than choosing an edge based on the given equation. This gives initial estimates of \begin{equation*}R(e)\end{equation*} for each edge and guarantees that the equation is well defined. At the end of each simulation run, if the simulation results in a reward of ρ, we update \begin{equation*}N(e)\end{equation*} and \begin{equation*}R(e)\end{equation*} for each edge \begin{equation*}e\end{equation*} in the chosen policy path, as given by

\begin{equation*}R(e) \leftarrow \frac{R(e) \cdot N(e) + \rho}{N(e) + 1}\end{equation*},

\begin{equation*}N(e) \leftarrow N(e) + 1\end{equation*}.

### H1N1 transmission model

Our model includes the 100 largest metropolitan areas in the United States, which we identified by aggregating Census Bureau Statistical Areas (CBSA) that share a common airport [10] [11]. We model movement among cities using both Census Bureau’s County-To-County Worker Flow Files [12] and the Bureau of Transportation Statistics Origin and Destination Survey for all quarters of 2007, which contains a 10 % sample of all itineraries between U.S. cities [13]. We assume that each latent traveler to a city has some chance of sparking an epidemic and that the probability of this happening is \begin{equation*}1 - \frac{1}{R_0 \cdot S}\end{equation*}, where \begin{equation*}S\end{equation*} is the fraction of susceptible individuals in the destination city’s population, as holds for a simple stochastic SIR model [14]. If there are \begin{equation*}N\end{equation*} infected travelers this week from city \begin{equation*}A\end{equation*} to city \begin{equation*}B\end{equation*} and the fraction of susceptibles in city \begin{equation*}B\end{equation*} is \begin{equation*}S\end{equation*}, then the model draws a binomial random variable from the distribution \begin{equation*}\operatorname{Binomial}(N, 1 - \frac{1}{R_0 \cdot S})\end{equation*} and creates that many new infected individuals in city \begin{equation*}B\end{equation*}. The number of infected travelers from city \begin{equation*}A\end{equation*} to city \begin{equation*}B\end{equation*} is calculated based on the travel data given as input, under the assumptions that symptomatic individuals do not travel and travelers are selected uniformly randomly from the population.

Within each city, disease transmission is modeled using a compartmental model with five compartments: susceptible, exposed, asymptomatic infectious, symptomatic infectious, and recovered (Fig. 2b). Progression from one compartment to another is governed by published estimates for H1N1 transmission and disease progression rates, as given in Table 1. Epidemics are initialized assuming that there are 100,000 cases of H1N1 in the United States (corresponding to the late June CDC estimate of over one million H1N1 cases [15]) distributed stochastically, proportional to city sizes. Assuming conservatively that universal H1N1 vaccine coverage will be achieved within 12 months, we terminate the simulations after 12 months or when all cases have recovered, whichever occurs first.

**Figure:**
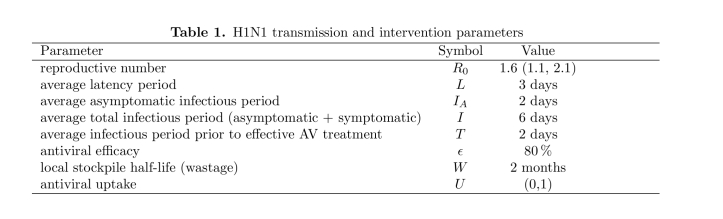
**Table 1.** H1N1 transmission and intervention parameters. References: reproductive number [16] [17]; average latency period [18]; average asymptomatic infectious period [16]; average total infectious period (asymptomatic + symptomatic) [18]; antiviral efficacy [19] [20] [21] [22] [23] [24] [25].

**Figure:**
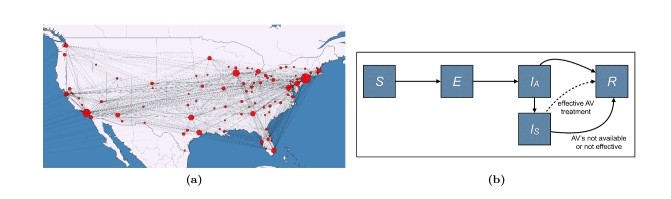
**Figure 2.** Disease Model. (2a) The U.S. network model for influenza transmission. Circle sizes represent numbers of inhabitants and line thickness represents the number of travelers between cities. (2b) Within-city compartmental model. When infected individuals progress from asymptomatic to symptomatic they seek treatment at a rate U (uptake) and receive treatment if antiviral courses are available locally. While disease transmission is a continuous process, antivirals are distributed once per day to those requiring treatment. Antivirals are assumed to be 80 % effective; and effectively treated cases immediately move to the recovered compartment. Untreated and ineffectively treated cases remain infectious until they recover naturally. The parameters of the compartmental model are described in Table 1.

## Antiviral policy actions

The model considers 11 possible antiviral stockpile actions every month over a twelve month period: distribution of 0, 1, 5, 10, 25 or 50 million courses apportioned either pro rata or proportional to current prevalence. We assume that, post distribution, unused courses decay through misuse or loss at a rate \begin{equation*}W\end{equation*}. Clinically, we assume that antivirals are 80 % efficacious at reducing disease and infectiousness [19] [20] [21] [22] [23] [24] [25]. If an infected individual resides in a jurisdiction with remaining distributed antivirals, then they receive appropriate treatment (i. e., access to medications within 24 hours of onset of symptoms) with an uptake probability of \begin{equation*}U\end{equation*}. Effectively treated infected cases (i. e., \begin{equation*}0.80 \times U\end{equation*}) are immediately moved from the infectious to the recovered compartment, consistent with early evidence of rapid decline in viral titers in treated H1N1 patients [26]. Consistent with current CDC antiviral guidance, we did not model the use of antivirals for large scale prophylaxis of susceptible populations in the absence of infection.

## Computational requirements

Each optimization is based on 48 hours of computation on the Linux Lonestar Cluster at the Texas Advanced Computing Center, which offers a peak performance of 10.7 GFLOPS per optimization process. Roughly 1,000,000 simulations can be done in this time; however, for this relatively small action space, the optimal policy is typically identified within six hours of computation.

## Results

Without antivirals, the expected cumulative number of cases after 12 months is almost 128 million. We found several optimized time-based intervention policies that decrease the spread of disease. Figure 3a shows the comparison of these strategies with a range of naive distribution policies in which fixed fractions of the antiviral stockpile are distributed monthly. Notably, one of these naive policies – monthly releases of 5 million regimens pro rata for 10 months – consistently performs as well as the optimized policies and better than other naive policies, particularly at uptake rates between 20 % and 60 %. Naive monthly distributions of less than 5 million regimens fail to meet the demand while larger monthly distributions result in greater wastage and rapid exhaustion of the stockpile (Fig. 3b).

**Figure:**
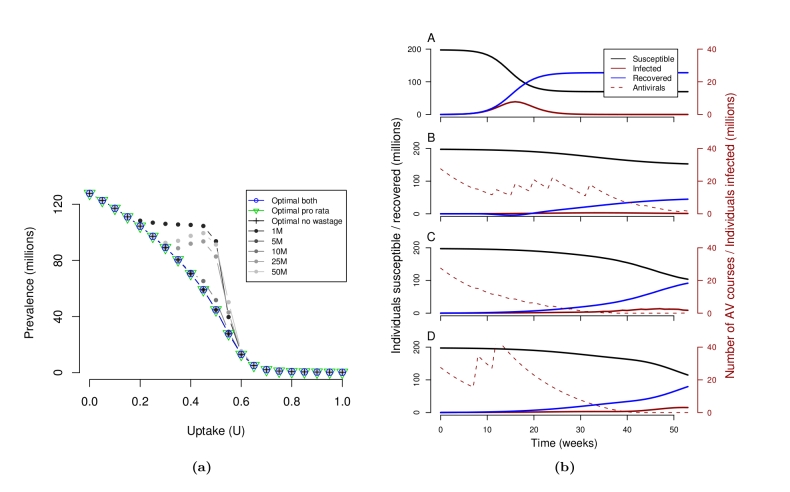
**Figure 3.** Optimizer performance. (3a) The optimal policies perform at least as well as the naive strategies of distributing fixed quantities each month until the stockpile is depleted. The four best policies are completely overlapping in the graph: the optimal policy when there is no wastage, the optimal pro rata policy, the optimal policy combining both prevalence-based and pro rata distributions, and the simple strategy of distributing 5M courses per month for ten months. In contrast, the other simple strategies perform poorly for many uptake values. (3b) Predicted prevalence curves at an uptake of 50 %. From top to bottom: without intervention, with the optimal intervention policy, releasing 1 million courses every month, releasing 25 million courses in each of the first two months. The dashed lines represent the total number of antivirals distributed and available for treatment. The 1 million per month policy does not release enough antivirals to meet demand; and the 25 million policy leads to a rapid depletion of antivirals, followed by a spike in the number of infections.

The optimal policy returned by the algorithm is sensitive to the levels of uptake (\begin{equation*}U\end{equation*}), but, remarkably, the optimal policies under high levels of wastage (loss of half of unused supplies every two months) are predicted to perform as well as optimal policies under no wastage (Fig. 3a). Under perfect conditions of no loss or misallocation of antivirals, then there is no reason to reserve the stockpile and the optimal strategies are always a series of large distributions early in the outbreak.

A mixed pro rata and prevalence-based distribution policy favored pro rata distributions, particularly for low to intermediate levels of uptake (Fig. 4a). This combined with the comparable performance of exclusively pro rata policies (Fig. 3a) suggests that prevalence-based distributions are probably unnecessary.

**Figure:**
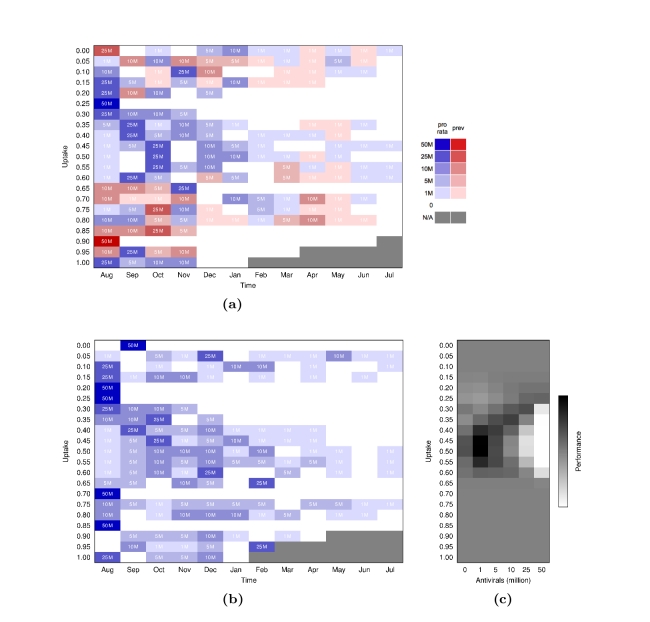
**Figure 4.** Optimal policies. (4a) Optimal policies combining prevalence-based (red) and pro rata distributions (blue). Each row gives the optimal sequence of actions for a given value of uptake. (4b) Optimal pro rata distributions. (4c) Performance of actions for the first (August) distribution. Darkness indicates how many times an action was visited during the optimization routine. At a modest and perhaps realistic 20 % uptake, the optimal combined strategy begins with distributing half of the remaining federal stockpile pro rata followed by monthly distributions of 10M proportional to prevalence, 10M pro rata, none, and 5M pro rata. The optimal pro rata policy is an initial distribution of the entire 50M regimens. (4c) indicates, however, that, at this uptake rate, all other distribution options are expected to perform similarly.

The optimal pro rata policies (Fig. 4b) vary considerably with uptake. At realistically low levels of uptake (between 10 % and 25 %) the optimal strategies are either a single distribution of the entire stockpile or a single distribution of half of the stockpile followed by a slow steady stream of smaller releases. For example, at 15 % uptake, the optimal policy is to distribute a twelve-month sequence of 25M, 1M, 10M, 10M, 1M, 0, 1M, 0, 1M, 0, 1M, and 0 courses. At higher levels of uptake, which are probably unlikely without a national campaign to increase rates of antiviral treatment for mild cases, the optimal strategies are a bit more complex, involving longer series of variable distributions. Between 35 % and 65 % uptake, the optimal strategies involve initial delays of a few months during which only small quantities are distributed. This delay prevents the wastage of antivirals that will go unused during the initial period when prevalence is relatively low. This delay strategy is even more pronounced when optimizing distributions for the entire epidemic period (Supplementary Information).

The optimization provides information about the relative performance of different actions. At uptake levels less than 25 %, the best strategies are expected to reduce the number of cases by at most 15 %. At even lower levels of uptake (less than 15 %), all initial (August) pro rata distributions perform equally well because only a very small proportion of the entire stockpile will ever be used to treat H1N1 (Fig. 4c). As uptake increases, there is a trade-off between meeting the increasing demand for treatment and risking wastage of courses that would otherwise be used to treat future cases of H1N1. Between 20 % and 30 % uptake, the benefits of increased coverage outweigh the consequent wastage, and thus the optimal action is a large release in August. Between 35 % and 60 %, wasted courses are more likely to have been used for future treatment, suggesting a steady distribution throughout the 12 month period (Fig. 4b). At uptake greater than 60 %, an overwhelming fraction of infected individuals seek treatment, and any distribution schedule effectively mitigates the epidemic for 12 months.

## Discussion

Since avian influenza H5N1 became a potential public health threat in 2003, public health agencies around the globe have been diligently planning for the next influenza pandemic. While the concerted response to H1N1 reflects this careful preparation, several expected and unexpeted events, including its apparent North American origin, the rapid overburdening of U.S. laboratory capacity, non-uniform testing and treatment policies among U.S. states, and delays in production of a viable vaccine, all reinforce the need for a dynamic and quantitative playbook for pandemic mitigation using pharmaceutical countermeasures.

By adapting an established algorithm to optimize disease mitigation policies, we advance from the traditional candidate strategy approach to rapid and systematic analysis of numerous policy options. This is just one of many possible optimization methods suitable for this purpose [27] [28] [29] [30]. Our choice of UCT was based on the insight that, with some careful modeling, disease intervention strategies can be nicely mapped onto policy trees and that this approach can be coupled to any stochastic epidemic model. This approach has performed successfully on large policy trees [31] and has favorable convergence properties [28]. In particular, it is guaranteed to converge on the optimal policy eventually, unlike simulated annealing and genetic algorithms. We do not claim that the optimized antiviral strategies (Fig. 4) are necessarily the best possible. Rather, we simply argue that in a reasonable amount of time, the model converged on policies that are predicted to perform equal to or better than obvious candidate policies, and that the resulting policies make intuitive sense.

Unexpectedly, our analysis suggests that the optimal distribution schedule for the U.S. national antiviral stockpile may be quite simple. A monthly distribution of 5 million regimens to states consistently matches or outperforms other policy options, regardless of the level of uptake or rate of misuse (Fig. 3a). Slight variations on this policy, for example, regular distributions of 1M or 10M courses are predicted to perform significantly worse at intermediate levels of uptake. Since there can be many optimal policies, the results of the optimization algorithm do not necessarily have the same simple structure as the 5M strategy. Our optimization allowed for the possibility of distributions proportional to prevalence, although such actions are not consistent with the current strategic national stockpile policy and would likely be both politically and logistically difficult. Notably, the results suggest that prevalence-based distributions are not expected to enhance the impact of antivirals.

The expected impact of this simple 5 million course monthly distribution schedule is highly sensitive to the rate of antiviral treatment (\begin{equation*}U\end{equation*}). From an ongoing study of H1N1 antiviral uptake in Milwaukee, preliminary estimates of the fraction of reported cases receiving treatment within 48 hours of developing symptoms are less than 20 % [32]. This suggests that we are likely in the range where all strategies perform equally poorly and are predicted to minimally mitigate transmission. Although treatment beyond 48 hours may not alter clinical course, there is some evidence that it may lead to a more rapid drop-off in viral shedding thus reducing transmission [26]. Thus public health measures to increase the usage of antivirals for H1N1 have the potential to slow transmission prior to the availability of H1N1 vaccine, but the impact of such measures will critically depend on the Strategic National Stockpile distribution schedule. Although antivirals may not reduce transmission at current levels of uptake, they can significantly reduce morbidity and mortality associated with H1N1 when used to treat potentially severe cases.

Our analysis did not consider the threat of antiviral resistance. Currently circulating strains of seasonal influenza have acquired resistance to oseltamivir [33] and there is evidence that H1N1 has exhibited this resistance as well [34]. We also did not incorporate the use of antivirals for prophylaxis, the future availability of vaccines, simultaneous use of NPI’s like school closures, or the option of targeting the stockpile towards particular demographic groups, all of which are likely important and may influence the optimal policy.

From rapid genetic sequence analysis to automated syndromic surveillance systems, public health emergency response is rapidly improving in technical capabilities both in the U.S. and worldwide; the rapid response to and characterization of the novel pandemic influenza A (H1N1) virus is a testament to this. However, planning the policies of public health response to such identified and emergent threats remains a highly non-quantitative endeavor. We present here a policy optimization approach that is highly modular and can be easily adapted to address multiple additional issues. Our hope is that the quantitative methods will assist clinical experts in developing effective policies to mitigate H1N1 using a combined arsenal of vaccines, antivirals and NPI’s. Specifically, a very similar analysis can be used at the international level to optimize global allocation of the WHO's limited antiviral stockpile to resource-poor countries. One can substitute any stochastic model of disease transmission, at any scale, for our national-scale, U.S. H1N1 model. In addition, while the optimization algorithm is particularly well suited for time-based interventions, any well-behaved policy space can be used [28]. The approach should thereby facilitate a more comprehensive consideration of pandemic policy options, and will perhaps confirm the efficacy of the current policy or suggest more strategic options for the future [28]. The approach should thereby facilitate a more comprehensive consideration of pandemic policy options, and will perhaps confirm the efficacy of the current policy or suggest more strategic options for the future.

### Supplemental Analysis   Supplemental Visualization

## Acknowledgments

The findings and conclusions in this report are those of the authors and do not necessarily represent the official position of the Centers for Disease Control and Prevention. The authors thank John Tegeris at BARDA for providing up-to-date information about the U.S. federal and state Strategic National Stockpiles of antivirals, the BARDA flu vaccine group for providing up-to-date estimates for the availability of H1N1 vaccines, the City of Milwaukee Health Department and the Harvard Center for Communicable Disease Dynamics for sharing unpublished data on the receipt of oseltamivir in Milwaukee. The authors acknowledge the Texas Advanced Computing Center (TACC) at The University of Texas at Austin (http://www.tacc.utexas.edu) for providing HPC resources that have contributed to the research results reported within this paper.

## Funding Information

This work was supported by grants to LM from NIH Models of Infectious Disease Agent Study (MIDAS) (U01-GM087719-01), the James S. McDonnell Foundation, and NSF (DEB-0749097) and grants to BP from CIHR (PTL-97125 and PAP-93425) and the Michael Smith Foundation for Health Research.

## Competing Interests

The authors have declared that no competing interests exist.
